# Immunological Impact of a Gluten-Free Dairy-Free Diet in Children With Kidney Disease: A Feasibility Study

**DOI:** 10.3389/fimmu.2021.624821

**Published:** 2021-06-02

**Authors:** María José Pérez-Sáez, Audrey Uffing, Juliette Leon, Naoka Murakami, Andreia Watanabe, Thiago J. Borges, Venkata S. Sabbisetti, Pamela Cureton, Victoria Kenyon, Leigh Keating, Karen Yee, Carla Aline Fernandes Satiro, Gloria Serena, Friedhelm Hildebrandt, Cristian V. Riella, Towia A. Libermann, Minxian Wang, Julio Pascual, Joseph V. Bonventre, Paolo Cravedi, Alessio Fasano, Leonardo V. Riella

**Affiliations:** ^1^ Renal Division, Brigham & Women’s Hospital, Harvard Medical School, Boston, MA, United States; ^2^ Servicio de Nefrología, Hospital del Mar, Barcelona, Spain; ^3^ Department of Pediatrics, Pediatric Nephrology Unit, Instituto da Criança, Hospital das Clínicas - University of São Paulo Medical School (USP), São Paulo, Brazil; ^4^ Center for Transplantation Sciences, Department of Surgery, Massachusetts General Hospital, Boston, MA, United States; ^5^ Center for Celiac Research and Treatment, Massachusetts General Hospital for Children, Harvard Medical School, Boston, MA, United States; ^6^ Experimental Therapeutics/Interventional Trials Center, Boston Children’s Hospital, Boston, MA, United States; ^7^ Center for Clinical Investigation, Brigham & Women’s Hospital, Boston, MA, United States; ^8^ Division of Nutrition, Instituto da Criança, Hospital das Clínicas - University of Sao Paulo Medical School, Sao Paulo, Brazil; ^9^ Department of Medicine, Boston Children’s Hospital, Harvard Medical School, Boston, MA, United States; ^10^ Renal Division, Beth Israel Deaconess Medical Center, Harvard Medical School, Boston, MA, United States; ^11^ Beth Israel Deaconess Medical Center Genomics, Proteomics, Bioinformatics and Systems Biology Center, Division of Interdisciplinary Medicine and Biotechnology, Beth Israel Deaconess Medical Center, Harvard Medical School, Boston, MA, United States; ^12^ Medical and Population Genetics Program, Broad Institute of Massachusetts Institute of Technology (MIT) and Harvard, Cambridge, MA, United States; ^13^ Renal Division, Department of Medicine, Icahn School of Medicine at Mount Sinai, New York, NY, United States; ^14^ Division of Nephrology, Massachusetts General Hospital, Harvard Medical School, Boston, MA, United States

**Keywords:** steroid resistance nephrotic syndrome, inflammation, diet, gluten-free, dairy-free

## Abstract

Kidney disease affects 10% of the world population and is associated with increased mortality. Steroid-resistant nephrotic syndrome (SRNS) is a leading cause of end-stage kidney disease in children, often failing standard immunosuppression. Here, we report the results of a prospective study to investigate the immunological impact and safety of a gluten-free and dairy-free (GF/DF) diet in children with SRNS. The study was organized as a four-week summer camp implementing a strict GF/DF diet with prospective collection of blood, urine and stool in addition to whole exome sequencing WES of DNA of participants. Using flow cytometry, proteomic assays and microbiome metagenomics, we show that GF/DF diet had a major anti-inflammatory effect in all participants both at the protein and cellular level with 4-fold increase in T regulatory/T helper 17 cells ratio and the promotion of a favorable regulatory gut microbiota. Overall, GF/DF can have a significant anti-inflammatory effect in children with SRNS and further trials are warranted to investigate this potential dietary intervention in children with SRNS.

## Introduction

Idiopathic nephrotic syndrome (INS) is the leading cause of nephrotic syndrome and a major cause of end-stage kidney disease in children (1). Approximately 80-90% of children with INS achieve complete remission with first-line steroid therapy and are classified as steroid-sensitive nephrotic syndrome (2). About 10-20% of patients do not respond to steroids [steroid-resistant nephrotic syndrome (SRNS)] or experience frequent relapses after withdrawal (steroid-dependent nephrotic syndrome) (3). In a subset of SRNS, causative genetic mutations in podocytes genes have been described (4). In idiopathic SRNS with an initial response to steroids, it is suggested that circulating factor(s) cause the podocyte injury and proteinuria. This is reinforced by the finding of a high rate of recurrence of disease soon after transplantation (3). The exact nature of this circulating factor is still unknown, but it is highly likely to be related to immune activation (5–8).

Despite potent immunosuppressive agents available, treatment of SRNS remains challenging due to low response rate to therapy (9, 10), significant side effects and high-risk of end-stage renal disease. Over the past 50 years, a few case reports have suggested the potential impact of dietary changes in controlling certain types of INS, in which the pathophysiology seems to be associated with immune system activation related to food sensitivities (11, 12). In particular, gluten and dairy restrictions have been associated with significant decrease of proteinuria in INS (13–16). The exact mechanism of this potential beneficial dietary intervention is still unknown, but several hypotheses have been proposed (11, 13–15). Food sensitivity is linked to immune cellular dysfunction (16–19) and exposure to sensitive foods may trigger the release of inflammatory factors or cytokines that could damage the podocytes (14). In addition, gluten-sensitive patients tend to have higher levels of zonulin, a modulator of intercellular tight junctions (20). This molecule opens intercellular tight junctions in the intestinal mucosa, increasing permeability to potentially toxic proteins produced by the microbiota (17, 18). Circulating zonulin might also have a direct effect on podocyte permeability (21), which has been shown in *in vitro* experiments (22). Therefore, zonulin levels could potentially serve as a biomarker to differentiate patients inclined to a response to gluten dietary restriction from those who are not.

Although several case reports suggest that a dietary restriction might reduce proteinuria, research studies with careful monitoring of the intervention are needed to determine the generalizability of these findings and to explore pathophysiological mechanisms and potentially new biomarkers. We aimed to investigate the immunological impact and safety of a combined gluten-free and dairy-free (GF/DF) diet in children with SRNS in the setting of a four-week summer camp, where the dietary intervention could be tightly controlled and monitored for compliance.

## Methods

### Study Design and Participants

This is a mechanistic, proof-of-concept prospective intervention study of a GF/DF diet on SRNS for four weeks at a summer camp (Orlando, Florida). The study protocol was recently described in detail (23). Briefly, patients aged 1 to 21-year-old with SRNS [biopsy-proven primary focal and segmental glomerulosclerosis (FSGS) or minimal change disease (MCD)] were eligible to participate. An estimated glomerular filtration rate (eGFR) >50 ml/min defined by bedside Schwartz formula (24) and urine protein creatinine ratio (UPCR) ≥1 g/g at the time of the enrollment were required to participate. SRNS was defined as persistent proteinuria after at least 3 months of therapy (steroids or another immunosuppressive drug). All the inclusion and exclusion criteria are presented in **Table S1**. To exclude patients with celiac disease, all baseline serum samples were serologically tested by tissue transglutaminase (tTG) IgA and deamidated gliadin peptide (DGP) IgG (**Table S2**). Whole exome sequencing analysis (WES) was performed to exclude all possible known mutations related to SRNS (**Table S3**) (4) and it did not reveal any known kidney-disease related mutations in our participants.

### Dietary Intervention

The diet was GF/DF and low-sodium (< 800 mg/day) for all the patients. During the study period, children were clinically monitored weekly. Meal plans were assessed for nutritional balance and compliance with the United States Department of Agriculture (USDA) Food and Nutrition Service My Plate guideline (25), adapted to each age group.

To ensure a GF/DF diet, NASPGHAN (North American Society for Pediatric Gastroenterology, Hepatology, and Nutrition) and CDHNF (Children’s Digestive Health and Nutrition Foundation) guidelines were followed (26). Even though the environment and the diet of the study were thoroughly controlled, the compliance of each participant to the GF diet at the camp was assessed once by a gluten urine dipstick (IVYDAL™) that is able to detect the presence of gluten in urine up to 3 days post-exposure (27) (**Table S4**).

All participants were started on a diet without added salt and fluids were not restricted. During the camp, the hydration state of each participant was checked frequently. Depending on symptoms (e.g. low blood pressure), and at the physician’s discretion, a maximum of 2,000 mg of salt per day was allowed. No sugar was used for meal preparations, while fresh vegetables and fruits were preferentially used with minimal use of processed foods. Children received calcium supplementation ranging between 700 mg and 1300 mg daily, based on the dietary reference intake per age group, developed by the USDA.

### Samples Collection and Outcomes

Saliva samples were collected at baseline for genetic testing. During the camp, random morning urine samples were collected weekly and 24h-urine samples were collected once (day 20). Venous blood was collected at two time points (day 0 and day 26) in EDTA-tubes (BD-367863; BD-366643) and serum-separating tubes (BD367986), the latter were centrifuged within 2 hours for immediate serum separation. Morning random urine, stool, 24h-urine, serum and blood were also collected after the camp (day 54). Stool was transferred into 2mL vials with and without RNA later. Urine, stool and serum samples were shipped on ice, EDTA-tubes in room temperature. All samples were shipped overnight to Boston and were processed within 36 hours from collection. Serum was aliquoted and frozen at -80 degrees Celsius. 300 uL EDTA-whole blood was used for analysis by flow cytometry, the remaining volume was used for peripheral blood mononuclear cell (PBMC)-isolation by a standard Ficoll procedure (28) and stored in liquid nitrogen until further analysis. Urine was centrifuged at 3,200 rpm for 5 minutes and stored in -80 degrees Celsius.

### Genetic Testing

DNA extraction from saliva, DNA sample quality assessment, DNA library preparation and WES were conducted at GENEWIZ, Inc. (South Plainfield, NJ, USA). Genomic DNA was extracted using the PureLink Genomic DNA extraction kit (ThermoFisher, CA, USA) per the manufacturer’s protocols. DNA was quantified using both Nanodrop 8000 and Qubit 2.0 Fluorometer (Life Technologies, Carlsbad, CA, USA). 50-60 ng of each sample was loaded on a 0.6% agarose gel to check sample integrity. Sequencing quality control was performed by FastQC and Trimmomatic (version 0.36) (29) before the reads were fed to Burrows-Wheeler Aligner (BWA, version 0.7.13-r1126) (30). Alignment was done by Maximum Exact Matches algorithm (BWA-MEM) against the human reference genome (GRCH38, hg38). After that, the variants were called according to the best-practices for use of the Genome Analysis Toolkit (GATK) by HaplotypeCaller algorithm (31, 32). Detected variants were compared to the kidney disease associated gene list extracted from Vivante et al. (33), which consisted of over 200 genes previously identified as causative of monogenic forms of kidney disease. The specific 39 genes linked to SRNS are represented in **Table S3**.

### Flow Cytometry Analysis

Immediately after arrival of the samples, fresh whole blood from each patient was used to analyze immune populations by flow cytometry (100 μL per antibody panel). EDTA was removed from blood by two washes with PBS 1x. Antibody (clone number in parenthesis) used are: Panel 1 Treg-Tfh: CD4 (OKT4), CD25 (B1.49.9, Beckman Coulter), CD127 (A019D5), CXCR5 (J252D4), PD-1(A17188B), CD8 (SK1); Panel 2 Tmem-Teff: CD4 (OKT4), CD8 (RPA-T8), CD45RA (HI100), CXCR3 (G025H7), CCR7 (G043H7), CCR6 (G034E3); Panel 3 Monocytes: CD14 (RMO52, Beckman Coulter), CD3 (UCHT1), CD11c (3.9), CD16 (3G8), HLA-DR (L243), CD56 (5.1H11), CD19 (HIB19); all antibodies from Biolegend^®^, San Diego, CA, US, unless otherwise noted. Antibody mixtures were added to the cells and were incubated for 30 min in room temperature. Subsequently, 1 ml of 1x lysing buffer (BD FACS Lysing solution) was added for 10 min. Cells were then washed with FACS buffer (PBS 1x, 2% FBS, 0.02% sodium azide) and immediately run at BD FACSCanto II flow cytometry. FlowJo^®^ software (FlowJo LLC, Ashland, OR, US) was used for analysis.

### Biomarkers

Frozen serum and urine were thawed for biomarker analysis. Serum was centrifuged at 15,000 x *g* for 10 min to remove potential lipids from the samples. Cytokine profiles were determined using the Human Cytokine/Chemokine Magnetic Bead Panel protocol from the MILLIPLEX^®^ MAP 23-Plex Kit from Millipore™ (HCYTOMAG-60K, Luminex^®^). The protocol was executed according to the manufacturers’ instructions; incubation was performed overnight for 16 h at 4°C. Plates were run on the Luminex 200^®^ machine and data were collected using the Luminex xPONENT^®^ software (v. 3.1). Analysis of the cytokine/chemokine mean fluorescent intensity (MFI) and conversion to concentration of the cytokine was performed using the Milliplex^®^ Analyst software (v. 3.5). Standard curves were verified and the coefficient of variation (CV) of duplicate wells were checked. Samples with a CV of more than 20% were excluded if the concentration of the particular analyte was not in the lower ranges of the standard curve. For samples with a value above/below bounds, the high/low bound was put in. Cytokines that showed above/below bounds values for more than half of the samples were excluded from analysis. In serum, in most samples the concentrations of IFN-g, IL-10, IL12-p40, IL-12p70, IL-13, IL-15, IL-4, IL-5, IL-6, IL-7, IL-9, IL-17a IL-1B, IL-2, MCP-3, CD40L and VEGF did not reach the lower detection bound or exceeded upper detection limits, and were therefore not further analyzed. In urine, the same applied for TNF-a, IL-10, IL-12p70, CD40L, IL-17, IL-9, IL-1B, IL-2, IL-5 and IL-7.

KIM-1, TNFR1, TNFR2, YKL40, and SuPAR were measured using in-house developed magnetic microbead based assay on a Luminex platform (BioPlex). Briefly, 30uL of serum/urine sample and recombinant protein cocktail (RnD system) were incubated with magnetic microbeads (Luminex Corp) that were coupled with captured antibodies for 1 h on an orbital shaker at 300 rpm. After incubation, beads were washed three times with Phosphate Buffered Saline with Tween (PBS-T) and incubated for another 1 h with corresponding detection antibodies (RnD systems). Beads were washed three times with PBS-T and incubated with SAPE-PE (Invitrogen) for 15 min. The signal from the fluorochrome, which is directly proportional to the amount of antigen bound at the micro-bead surface, was captured using the Bio-Plex 200 system (Bio-Rad).

### Proteomics

The SOMAscan^®^ is a high multiplex, high sensitivity aptamer-based immune like protein and biomarker discovery platform that can simultaneously quantify 1,310 proteins in the sera (34). 50uL of serum of each participant was used from day 0 and day 26 after being centrifuged at 10,000 rpm to remove lipids. Each assay was run along with calibration and normalization samples, according to the SomaLogic good laboratory practice quality system. Data from all samples passed quality-control criteria and were fit for analysis. Data was log-transformed to avoid violation of normality assumptions and tested by paired t-test. False discovery rate (FDR) was used to correct for multiple comparison with a cutoff of 0.1. Significant proteins after FDR correction were subsequently used for network enrichment analysis using MetaCore (v. 6.37, Clarivate Analytics) Enriched pathways were considered significant with a FDR <0.05. Heat maps were created using Morpheus (Morpheus, https://software.broadinstitute.org/morpheus).

### Microbiota Analysis

Total DNA from stool samples was isolated using Qiagen DNeasy powersoil extraction kits (Qiagen) following the manufacturer instructions. The hypervariable region V4 of the 16rRNA gene was amplified by PCR using 5Xprime master mix (Prime). We used barcoded 806 reverse primers and unique forward 515 primer (IDT). The amplification was confirmed by electrophoresis and the amplified products were purified with Quiaquick PCR purification kit (Qiagen). Samples were pooled together and sent to the MGH NGS Core facility to be sequenced on the Illumina MiSeq system using MiSeq v2 500 cycles reagent kit. The system sequenced a total of 250 paired-end cycles that allowed the maximum coverage of the amplicon. For the sequencing the following primers were used:

read 1 (TATGGTAATT GT GTGYCAGCMGCCGCGGTAA),
read 2 (AGTCAGCCAGCCGGACTACNVGGGTWTCTAAT)
index (AATGATACGGCGACCACCGAGATCTACACGCT)

Computational analysis on the 16s sequencing data was performed suing the QIIME2 software package. Low score quality reads (average Q < 25) were truncated to 240bp and filtered using *deblur* algorithm with default settings. *Mafft* was used to align the remaining high-quality reads to the reference library. Next, the aligned reads were masked to remove highly variable positions, and a phylogenetic tree was generated from the masked alignment using the FastTree method. Beta diversity metrics and Principal Component Analysis plots based on Jaccard distance were generated using default QIIME2 plugin. Taxonomy assignment was performed using *feature-classifier* method and naïve Bayes classifier trained on the Greengenes 13_8 99% operational taxonomic units (OTUs).

### Zonulin Measurement

Circulating zonulin was measured by competitive enzyme-linked immunosorbent assay (ELISA) (Immundiagnostik AG^®^, Bensheim, Germany) according to the manufacturer recommendations in serum samples for each patient over time. Intra-assay coefficient of variation was 6.5%.

### Statistical Analysis

Continuous variables are presented as mean (± standard deviation) or median (interquartile range), according to their distribution (assumption of normality assessed by graphics due to small sample size). Categorical parameters are shown as number of patients with associated percentages. Comparison of paired data between day 0 and day 26 (clinical outcome, flow cytometry, biomarkers, microbiota) was conducted using Wilcoxon signed-rank test. SOMAscan data was log-transformed and compared by paired t-test. Multiple comparison in all analysis was corrected for by FDR <0.1. Comparisons between responders versus non-responders (baseline zonulin levels) were analyzed by Mann-Whitney U test. Statistical analyses were performed using Prism 5.0b software (GraphPad software, Inc) and Stata software (StataIC-15, StataCorp LLC).

**Figure 1:**
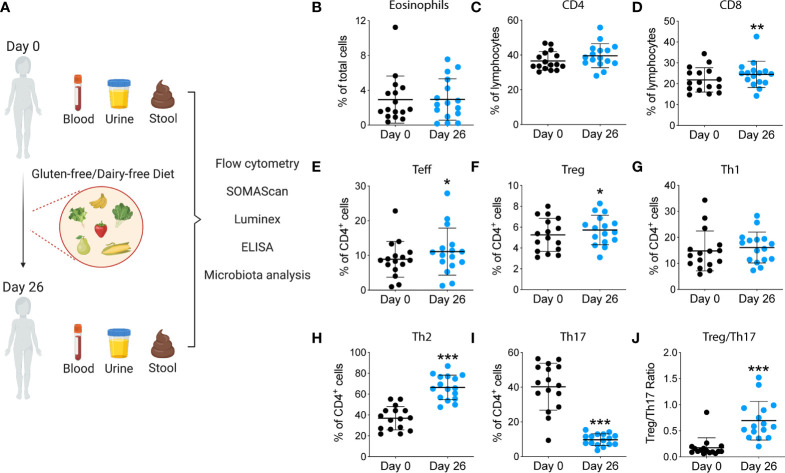
Study design and immunological analyses performed during the summer camp. **(A)** Blood, urine and stool were collected at the beginning of the summer camp. Participants underwent a GF/DF diet for 4 weeks. After that, samples were collected again and immune cells, proteins and microbiota were analyzed. **(B-J)** Changes in immune cell populations after GF/DF intervention. Flow cytometric analysis of fresh blood samples obtained from children at day 0 and day 26 during the camp, including eosinophils **(B)**, CD4+ T cells **(C)**, CD8+ T cells **(D)**, effector T cells **(E)**, regulatory T cells **(F)**, Th1 cells **(G)**, Th2 cells **(H)**, Th17 cells **(I)** and Treg/Th17 ratio **(J)**. Wilcoxon matched pair test was used to assess differences within immune subpopulations before and after gluten and dairy removal. Asterisks show significant values after correction for multiple testing by FDR<0.1. CM, central memory; Teff, T effector; T reg, T regulatory; *p<0.05; **p<0.01; ***p < 0.001.

**Figure 1A** represents the study diagram, with the diet intervention, samples collection and analyses performed. The cartoon was created with BioRender.com.

## Results

### Demographics

Thirty-eight patients were initially screened for the study. Seventeen candidates fulfilled all inclusion criteria during the screening period and provided consent for the summer camp. One patient experienced a medical complication prior to the camp and could not attend the camp. Finally, sixteen patients were included in the study. After analysis of baseline blood and urine samples at day 0 in the camp, four patients turned out to have less than 1-gram proteinuria per day; one patient had an eGFR of less than 50ml/min/1.73m^2^. Mean age was 7 ± 5.3 years and 50% of patients were female (Baseline characteristics summarized on **Table 1**). Ten of sixteen participants (63%) had histological diagnosis of FSGS, while the remaining 6 participants had MCD on initial biopsy. Median time since diagnosis to the camp was 34.5 months. Median proteinuria at day 0 was 5.1 g/g. Detailed demographic and clinical features of each patient are shown in **Table S5**.

**Table 1:** Baseline characteristics of participants.

	Children with SRNS (n=16)
Age, *years– mean ( ± SD)*	7.0 ± 5.3
Female gender *– n (%)*	8 (50)
Ethnicity *– n (%)*	
*Caucasian*	7 (44)
*African-American*	2 (13)
*Hispanic*	3 (19)
*Multiracial*	4 (25)
Histological diagnosis *– n (%)*	
*Minimal change disease*	6 (38)
*Focal Segmental Glomerulosclerosis*	10 (63)
Family history of kidney disease *– n (%)*	3 (19)
Age on onset of disease, *years – mean (* ± *SD)*	7.4 ± 5.3
Time diagnosis to Camp D0, *months – median [IQR]*	35 [12-60]
Time diagnosis to biopsy, *months – median [IQR]*	3 [1-4]
Previous second line IS therapy^*^ *– n (%)*	14 (88)
Previous third line IS therapy** *– n (%)*	9 (56)
Previous albumin infusion *– n (%)*	9 (56)
Previous partial or complete remission *– n (%)*	7 (44)
Number of anti-hypertensive drugs per participant *– n (%)*	1.38 ± 0.6
Patients on anti-proteinuric drugs – *n (%)* (ACEI/ARB/spirinolactone)	13 (83)
Patients on diuretics *– n (%)*	8 (50)
Serum creatinine *– median [IQR]*	0.54 [0.28-0.71]
eGFR** *– median [IQR]*	111 [91-154]
UPCR *– median [IQR]*	5.1 [0.9-9.0]
Serum albumin *– median [IQR]*	2.3 [1.8-2.8]
Systolic BP, *mmHg – mean ( ± SD)*	105 ± 11
Diastolic BP, *mmHg – mean ( ± SD)*	70 ± 9
Weight, *kg – median [IQR]*	36.8 [24.4-46.6]
Edema *– n (%)*	
*No*	9 (56)
*Mild*	3 (19)
*Moderate or severe*	4 (25)
Allergy susceptibility or atopy *– n (%)*	
*Respiratory*	4 (25)
*Cutaneous*	1 (6)
*Both*	2 (13)
History of infectious complication *– n (%)*	
*Mild frequent*	8 (50)
*Severe*	5 (31)
History of metabolic complications *– n (%)*	
*Weight gain*	5 (31)
*Growth retardation*	2 (13)
History of other complications *– n (%)*	
*Cataract*	3 (19)
*Bone disorders*	2 (13)
History of chronic diarrhea *– n (%)*	2 (13)
Prematurity *– n (%)*	
*Born before 39 weeks GA*	6 (38)
*Born before 37 weeks GA*	2 (13)

Values represent frequency (percentage), mean ± standard deviation or median [interquartile range]. SRNS, steroid-resistant nephrotic syndrome; eGFR, estimated glomerular filtration rate; UPCR, urine protein/creatinine ratio; BP, blood pressure; GA, gestational age; IS, immunosuppressive.

*Calcineurin inhibitors or antimetabolite agents.

**Rituximab or alkylating agents.

***Defined by bedside Schwartz formula (GFR = [Height in cm] x 0.413/serum creatinine mg/dL).

### Dietary Intervention Was Associated With a Reduction in Th17 Cells and Inflammatory Markers

**Figure 2:**
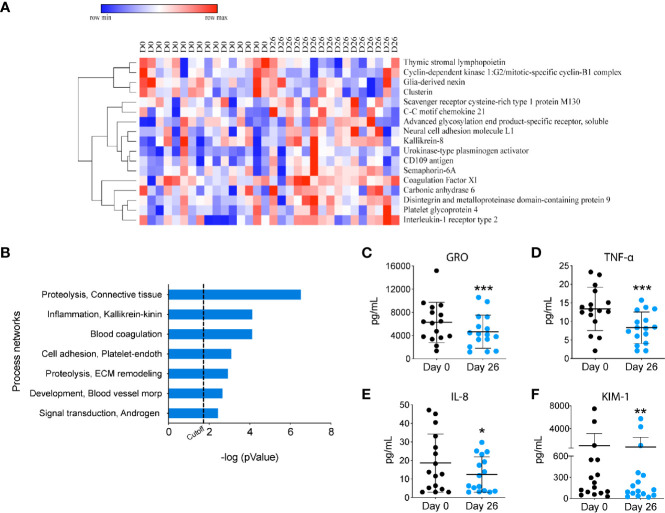
Proteomic analysis of circulating biomarkers upon GF/DF intervention. **(A)** Heatmap of the top 17 proteins that changed in all participants during the camp, measured by SOMAscan. **(B)** Process network enrichment analysis of the top 17 proteins using Metacore. **(C-F)** Changes in serum biomarkers. Serum levels of cytokines and kidney biomarker were measured by Luminex, including GRO **(C)**, TNFα **(D)**, IL-8 **(E)** and KIM-1 levels **(F)**. Wilcoxon matched pair test was performed to assess differences between before and after GF/DF intervention. Asterisks show significant values after correction for multiple testing by FDR<0.1. KIM, Kidney Injury Molecule; GRO, growth-regulated oncogene; *p<0.05; **p<0.01; ***unadjusted p<0.001.

To investigate the impact of a GF/DF diet on the immune function, we assessed specific immune cells subpopulations implicated in INS pathogenesis by flow cytometry and serum biomarkers by Luminex and SOMAscan. We observed a significant (albeit small) increase in CD8^+^ T cell population (**Figure 1D**) and an increase in CD4^+^ T effector cells upon dietary intervention at day 26 compared to baseline (**Figure 1C**). We also found a significant decrease in T helper 17 (Th17) subpopulation (**Figure 1I**) with a parallel increase in Th2 (**Figure 1H**). Importantly, GF/DF diet was associated with significantly increased regulatory T cells (Tregs, **Figure 1F**). Treg/Th17 ratio increased significantly in the population after dairy and gluten removal from the diet (**Figure 1J**). No changes in eosinophils, (**Figure 1B**), T effector cells (**Figure 1E**), T helper 1 (**Figure 1G**), granulocytes, T follicular helper cells, dendritic cells, B cells or natural killer cells were found (**Figure S1**). Analysis of proteomics in serum by SOMAscan®, which quantifies 1,310 proteins using an aptamer-based technology, identified 17 top-proteins that significantly changed during the camp in the participants (**Figure 2A**). Fourteen out of the 17 proteins are involved in the immune response, which is reflected in the process network analysis (**Figure 2B**). Biomarker analysis by Luminex showed a significant decrease in pro-inflammatory cytokines GRO (unadjusted p<0.001, FDR 0.003), TNF-α (unadjusted p<0.001, FDR 0.001) and IL-8 (unadjusted p=0.04, FDR 0.062) in serum after implementation of the diet (**Figures 2C–E**. We also observed a decreased concentration of kidney tubular injury marker KIM-1 (unadjusted p=0.002, FDR 0.008, **Figure 2F**), whereas other analyzed proteins did not show a significant difference (data not shown). Among urinary biomarkers, no significant differences were observed (data not shown). In sum, the summer camp with GF/DF diet had significant immune consequences both at cellular and protein levels by reducing inflammation and promoting Tregs.

### GF/DF Diet Promotes a Regulatory Gut Microbiome

**Figure 3:**
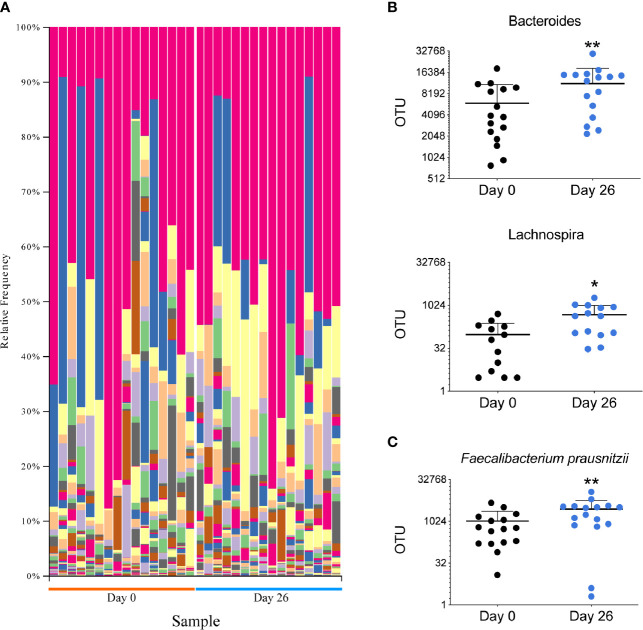
Analysis of the microbiota in all participants before and after GF/DF intervention. **(A)** Taxa plots representing the relative frequency of bacteria at genus level in fecal samples collected before and after the camp. Each color represents a bacterial genus and each column represents a patient sample. **(B)** Difference in fecal relative abundance of genera *Bacteroides* and *Lachnospira* before and after the camp. **(C)** Fecal relative abundance of species *Faecalibacterium prauznitzii* before and after the camp. Wilcoxon matched pair test was performed to assess differences in relative abundance of operational taxonomic units (OTUs), asterisks show significant values after correction for multiple testing by FDR<0.1. *unadjusted p<0.05; **unadjusted p<0.01.

The microbiota plays a major role in the induction and function of the host immune system. The diet can have a major influence in the characteristics and diversity of the microbiota. To assess the impact of GF/DF diet on the microbiota, we performed 16S rRNA gene analysis on DNA extracted from fecal samples at day 0 and at day 26 (**Figure 3A**). Taxonomic composition analysis showed a significant increased fraction of *Bacteroides*, *Lachnospira* and *Faecalibacterium* in the samples collected after the camp as compared to the ones collected pre-camp (**Figure 3B**). At the species level, an increase of *Faecalibacterium prausnitzii* was observed (**Figure 3C**). Overall, the summer camp with GF/DF diet promoted a favorable microbiome modification with potential immune regulatory phenotype.

### Clinical Outcomes Upon Dietary Intervention

Despite the pilot nature of our study, two out of 16 participants achieved complete remission in proteinuria after 4 weeks on GF/DF diet. Both participants experienced recurrence in proteinuria after returning to their previous unrestricted diet (documented by urine dipstick), after which they immediately went back to a GF/DF diet, achieving again a sustained remission in proteinuria. In all other participants, we did not observe any significant changes in terms of kidney function, proteinuria or serum albumin levels after GF/DF diet.

**Figure 4:**
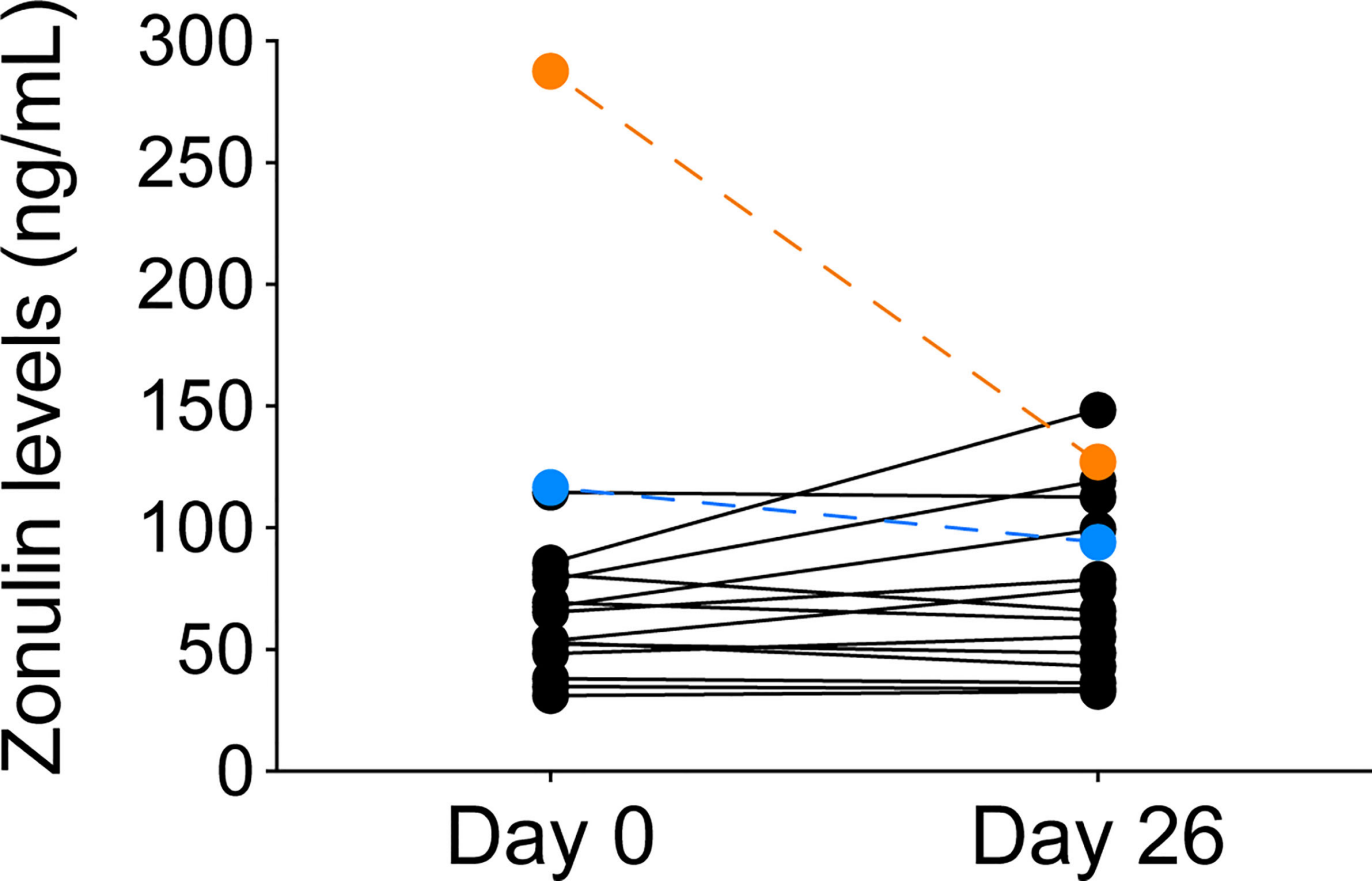
Zonulin levels before and after GF/DF diet implementation in all participants. Serum zonulin levels from serum samples obtained from children at day 0 and day 26 during the camp were determined by ELISA. Responders to the GF/DF intervention are shown in orange (patient 1) and blue (patient 6).

Zonulin, a circulating protein upregulated in gluten sensitivity, may affect gut permeability, and directly or indirectly alter podocyte tight junctions, a major barrier that prevents protein leakage into the urine. Baseline serum zonulin levels of the two responders were higher than the remaining cohort, and their values decreased after the dietary intervention (**Figure 4**). Other participants had lower baseline levels and had less variability during the camp. The difference in baseline zonulin levels between responders and non-responders were 202 ± 120.9 compared to 62.4 ± 21.9 ng/mL, respectively. With the exception of one patient, zonulin levels over 106.3 ng/mL at baseline (mean + 2 SD of non-responders) differentiated responders from non-responders.

Clinical parameters as blood pressure (BP) and weight were monitored weekly. No significant changes were observed in BP during the camp, with a mean of 105/70 at baseline and 108/62 after four weeks of GF/DF diet (**Figure S2A**). Weight remained also stable, although children experienced a decrease in weight during the first week, recovering to baseline during subsequent weeks (**Figure S2B**). Children started with a diet without added salt but amounts of 0.5 to 2 grams per day were reintroduced in seven patients due to symptoms (mainly fatigue) and low BP. Sodium intake was calculated with 24h urine sodium excretion during camp and after their return to their normal diet at home (**Figure S3**). Six patients decreased or stopped anti-hypertensive drugs during camp (four of these were diuretics). Conversely, two patients needed an increase in diuretic dose during the camp due to edema. Seven patients started the camp with edema (four of them moderate-to-severe), while only four patients presented with mild edema at the last day of the camp.

## Discussion

In this mechanistic, proof-of-concept, prospective study, we investigated the effect of GF/DF diet intervention over four weeks in children with SRNS in a summer camp setting. All participants had a decrease in inflammatory biomarkers by the end of the dietary intervention period. Regarding clinical outcomes, two out of sixteen children experienced a reduction in urine protein/creatinine ratio and had a recurrence of proteinuria once re-exposed to an unrestricted diet. These two children were found to have higher baseline serum zonulin levels than the remaining cohort. Overall, the dietary intervention was safe and not associated with any complication.

Environmental exposures such as the diet can have a significant impact in the immune system (35) and multiple studies have demonstrated an association between Th17 cells, inflammation and auto-immune disease (35–39). In patients with INS, increased levels of Th17 have also been observed (40–42). We found a significant reduction in Th17 cells in our participants after implementing the diet, and without any immunosuppression dose modification. By the end of the camp, they experienced a 4-fold increase in the Treg/Th17 ratio. A skewed ratio towards Th17 has also been related to autoimmune diseases, including INS in children (43, 44), and its dynamic regulation may be influenced by gut microbiome (45, 46). Due to the design of our study, we cannot assess whether the reduced proportion of Th17 cells was caused by specific changes in the diet such as reduced sodium intake (36, 38, 47, 48) or other factors such as the removal of gluten or dairy from the diet. Another indicator of reduced inflammation after GF/DF diet were the lower levels of TNF-α, GRO and IL-8 across all participants. Several cytokines/chemokines have been related to INS in animal as well human studies (49). TNF-α was found to be elevated in serum of patients with SRNS (50–52) and FSGS (53). Lastly, the more comprehensive SOMAscan^®^ analyses of the proteomic profile showed that the main proteins that changed were involved in inflammation and immune response.

The microbiome results showed differing taxonomic composition in all participants following the implementation of the GF/DF diet. There was an increased abundance of *Bacteroides, Lachnospira* and *Faecalibacterium* at the genus level, and an increase in *Faecalibacterium (F.) prausnitzii* at the species level. *F. prausnitzii* is one of the most abundant bacterial species found in healthy adults, and a reduction of this particular bacteria has been linked to different (auto-immune) intestinal disorders (54). *F. prausnitzii* has also been shown to be important in the Th17/Treg balance, by reducing Th17 cell differentiation and its potential beneficial effect in the treatment of inflammatory bowel disease (55, 56). There is some evidence that low levels of butyric acid, produced by among others *F. prausnitzii*, may play a role in relapsing INS (57), although larger studies are needed to confirm this. The increase of *F. prausnitzii* in our participants is in concordance with the reduced Th17 population observed and might be beneficial even in the absence of a reduction in proteinuria.

Zonulin secretion is increased in gluten-sensitive patients and it could play a role in the pathophysiology of SRNS through a direct effect on podocytes (22) or indirectly by enhancing gut permeability to potentially toxic (or immunogenic) proteins produced by the microbiota (17, 21, 22). Indeed, recent data demonstrated that zonulin levels were increased in children with INS (58) and we observed in our study that two children that experienced complete remission after GF/DF implementation had higher zonulin levels before the intervention and the level decreased at week 4. Although the exact mechanisms responsible for the proteinuria remain uncertain, one hypothesis is that exposure to sensitive antigens may trigger inflammatory mediators that could systemically lead to podocyte injury (14). Therefore, dietary intervention might be a complementary therapeutic tool to other immunosuppressive drugs in a subset of children with SRNS, improving the prognosis by achieving complete/partial remission, although studies with a control group and larger number of patients are needed to confirm this potential benefit. Whether zonulin levels could discriminate patients who would benefit of a GF diet remains uncertain, although there is a clinical trial ongoing focused on answering that specific question (59).

This pilot trial presents with several limitations. First, the study sample size is modest, and the patient recruitment and camp-structured study entail a selection bias, so consequently, the cohort might not reflect the diversity of children with SRNS. Secondly, there is no control group. This is a proof-of-concept study in which we aimed to assess the feasibility of a larger trial. Furthermore, the time of intervention could be too short for some children, even though most patients with SRNS who responded to the dietary intervention showed a quick reduction in proteinuria within one week. Finally, fourteen out of sixteen children had received other immunosuppressant agents (besides steroids) before the camp, and this might have an impact in the results (despite no changes in immunosuppression within 2 months of summer camp). While T cell dysregulation has been proposed to have a potential role is FSGS (1, 60), it is possible that B cells could also have a complementary role. Although we did not observe any significant changes in the proportion of B cells upon intervention, future studies will need to better characterize potential B cell subsets that may be involved in the pathogenesis of FSGS, in particular in SRNS. Nonetheless, we believe the overall pattern of the diet that we deployed had a major impact in the immune system, requiring further investigation in patients with kidney disease as well as other auto-immune diseases.

Overall, this is the first trial designed to assess the effect of a dietary intervention in SRNS, using an innovative study design in a summer camp (23). The camp allowed tight control of the intervention but simultaneously provided a friendly environment where children and families could meet and share their experiences. While we did not formally assess the psychosocial benefit of the summer camp, it was clearly visible for the patients, family members and research staff. For a dietary intervention trial, the camp offers the opportunity of introducing and complying to a GF/DF diet in a structured way, eliminating difficulties that could occur at home, such as accidental intake of gluten/dairy due to a lack of diet knowledge, temptations to eat restricted foods and the need to shop and prepare specific food. Lastly, this design ensures an easy and standardized way of sample collection, as conditions for all participants and samples are equal, and provides the opportunity of close medical supervision and detailed follow-up. We acknowledge the potential bias that could emerge in real-world practice. Following a strict diet is not easy to accomplish, especially in children, and it is associated with greater financial expenses (61, 62). However, the risk/benefit of avoiding specific foods over following immunosuppression treatment for the rest of their lives, inclines the balance to the effort that the diet might imply.

In conclusion, we present the first pilot clinical trial that has evaluated the impact of a restrictive GF/DF diet on proteinuria in children with SRNS in a controlled dietary environment over the course of a summer camp. We observed a decrease in the inflammatory status of these children. Besides that, two out of sixteen children achieved complete remission and high zonulin level was predictive of responsiveness, therefore may be further investigated as a potential biomarker for dietary intervention. A summer camp is a feasible way to implement dietary interventions in children and assess its short-term effect. Although this is a proof-of-concept study, our data provide a strong rational for a larger randomized study aimed to validate these findings.

## Data Availability Statement

The original contributions presented in the study are publicly available. This data can be found here: BioProject, PRJNA732410.

## Ethics Statement

The studies involving human participants were reviewed and approved by 2017P000615/PHS. Written informed consent to participate in this study was provided by the participants’ legal guardian/next of kin.

## Author Contributions

LR initiated the study. LR, MP-S, AU, JL, AW and AF designed, organized and prepared the study and summer camp. MP-S, AU, JL, NM, AW, VK, AF and LR were present at the camp. PC, PCu, LK, KY and CF were involved in nutritional aspects of the camp and meal preparation. MP-S, AU, JL, NM, LR and VK were involved in clinical data collection and sample collection, shipping and processing. MP-S and NM performed flow-cytometric analysis. VS performed the kidney biomarker assays, while JB helped with interpretation. CR and MW performed genetic analysis with verification by FH. Proteomics were performed by AU, TL and TB. Microbiota analysis was performed by GS, AU and AF. MP-S, AU, JL and LR wrote the manuscript. All authors contributed to the article and approved the submitted version.

## Funding

This study was funded by philanthropic donation. LR is supported in part by the Harold and Ellen Danser Endowed/Distinguished Chair in Transplantation at Massachusetts General Hospital.

## Conflict of Interest

FH is a cofounder of Goldfinch-Bio.

The remaining authors declare that the research was conducted in the absence of any commercial or financial relationships that could be construed as a potential conflict of interest.
