# Corrigendum: Immunological Impact of a Gluten-Free Dairy-Free Diet in Children With Kidney Disease: A Feasibility Study

**DOI:** 10.3389/fimmu.2021.798560

**Published:** 2021-11-02

**Authors:** María José Pérez-Sáez, Audrey Uffing, Juliette Leon, Naoka Murakami, Andreia Watanabe, Thiago J. Borges, Venkata S. Sabbisetti, Pamela Cureton, Victoria Kenyon, Leigh Keating, Karen Yee, Carla Aline Fernandes Satiro, Gloria Serena, Friedhelm Hildebrandt, Cristian V. Riella, Towia A. Libermann, Minxian Wang, Julio Pascual, Joseph V. Bonventre, Paolo Cravedi, Alessio Fasano, Leonardo V. Riella

**Affiliations:** ^1^ Renal Division, Brigham & Women’s Hospital, Harvard Medical School, Boston, MA, United States; ^2^ Servicio de Nefrología, Hospital del Mar, Barcelona, Spain; ^3^ Department of Pediatrics, Pediatric Nephrology Unit, Instituto da Criança, Hospital das Clínicas - University of São Paulo Medical School (USP), São Paulo, Brazil; ^4^ Center for Transplantation Sciences, Department of Surgery, Massachusetts General Hospital, Boston, MA, United States; ^5^ Center for Celiac Research and Treatment, Massachusetts General Hospital for Children, Harvard Medical School, Boston, MA, United States; ^6^ Experimental Therapeutics/Interventional Trials Center, Boston Children’s Hospital, Boston, MA, United States; ^7^ Center for Clinical Investigation, Brigham & Women’s Hospital, Boston, MA, United States; ^8^ Division of Nutrition, Instituto da Criança, Hospital das Clínicas - University of Sao Paulo Medical School, Sao Paulo, Brazil; ^9^ Department of Medicine, Boston Children’s Hospital, Harvard Medical School, Boston, MA, United States; ^10^ Renal Division, Beth Israel Deaconess Medical Center, Harvard Medical School, Boston, MA, United States; ^11^ Beth Israel Deaconess Medical Center Genomics, Proteomics, Bioinformatics and Systems Biology Center, Division of Interdisciplinary Medicine and Biotechnology, Beth Israel Deaconess Medical Center, Harvard Medical School, Boston, MA, United States; ^12^ Medical and Population Genetics Program, Broad Institute of Massachusetts Institute of Technology (MIT) and Harvard, Cambridge, MA, United States; ^13^ Renal Division, Department of Medicine, Icahn School of Medicine at Mount Sinai, New York, NY, United States; ^14^ Division of Nephrology, Massachusetts General Hospital, Harvard Medical School, Boston, MA, United States

**Keywords:** steroid resistant nephrotic syndrome, inflammation, diet, gluten-free, dairy-free

An author name was incorrectly spelled as “Cravedil”. The correct spelling is “Cravedi”.

The authors apologize for this error and state that this does not change the scientific conclusions of the article in any way. The original article has been updated.

## Publisher’s Note

All claims expressed in this article are solely those of the authors and do not necessarily represent those of their affiliated organizations, or those of the publisher, the editors and the reviewers. Any product that may be evaluated in this article, or claim that may be made by its manufacturer, is not guaranteed or endorsed by the publisher.

