# Supplementary material for: Immunological Impact of a Gluten-Free Dairy-Free Diet in Children With Kidney Disease: A Feasibility Study
Source: Front Immunol. 2021 Jun 2;12:624821. doi: 10.3389/fimmu.2021.624821 (PMC8208082; doi:10.3389/fimmu.2021.624821)
Supplement: Supplementary file 1 [file DataSheet_1.docx]

Supplementary Material

# Supplementary Figures and Tables

## Supplementary Tables

**Table S1.** Genie study inclusion and exclusion criteria.

| **Inclusion criteria** | **Exclusion criteria** |
| --- | --- |
| 1. Male or female aged 1-21 years-old 2. Willing and able to provide written informed consent signed by patient if 21 years-old, or parent/guardian willing in any other cases, to comply with dietary changes 3. SRNS defined as persistent nephrotic-range proteinuria after at least 12 weeks of steroid therapy (starting with 60 mg/m^2^/day or 2mg/kg/day to a maximum of 60mg/day for 4-6 weeks and following as alternate-day medication)* 4. Biopsy-proven FSGS or MCD 5. eGFR > 50 ml/min defined by bedside Schwartz formula (GFR = [Height in cm] x 0.413/serum creatinine mg/dL) 6. UPC ratio ≥ 1g/g at screening with 2 different samples collected within the 3 months before camp 7. Concomitant use of ACEi/ARB at stable doses for at least 2 weeks before camp 8. No changes in immunosuppression for the previous 2 months | 1. Kidney disease other than FSGS or MCD 2. Patient with known gluten or milk allergy, proved by immunological tests 3. Patient with known pathogenic podocyte genetic mutation |

ACEi, angiotensin conversion enzyme inhibitors; ARB, angiotensin II receptors blockers; eGFR, estimated glomerular filtration rate; FSGS, focal and segmental glomerulosclerosis; MCD, minimal change disease; SRNS, steroid resistant nephrotic syndrome; UPC, urine protein/creatinine

**KDIGO Clinical Practice Guideline for Glomerulonephritis. Kidney International Supplements (2012) 2, 143–153; doi:10.1038/kisup.2012.13*

**Table S2.** Celiac disease serology at baseline (on a regular diet).

|  | **tTG IgA** | **DGP IgG** |
| --- | --- | --- |
| Patient 01* | 1.9 | 2.8 |
| Patient 02 | 8 | 2.8 |
| Patient 03 | 1.9 | 2.8 |
| Patient 04 | 1.9 | 2.8 |
| Patient 06* | 1.9 | 2.8 |
| Patient 07 | 1.9 | 2.8 |
| Patient 08 | 1.9 | 2.8 |
| Patient 09 | 2.2 | 2.8 |
| Patient 10 | 2.5 | 2.8 |
| Patient 11 | 1.9 | 2.8 |
| Patient 12 | 1.9 | 2.8 |
| Patient 14 | 1.9 | 2.8 |
| Patient 15 | 1.9 | 2.8 |
| Patient 16 | 3.8 | 2.8 |
| Patient 17 | 1.9 | 2.8 |
| Patient 18 | 1.9 | 2.8 |

*Patients 1 and 6 were the responders

DGP IgG, IgG antibodies against deamidated gliadin peptides (U); tTG IgA, IgA antibodies against tissue transglutaminase (UI/mL)

**tTG IgA interpretation**: <20.0 U/mL (negative); >20.0 U/mL (positive)

**DGP IgG interpretation**: <20.0 U (negative); >20.0 U (positive)

**Table S3.** Known mutations related to SRNS analyzed by whole exome sequencing analysis.

| **Autosomal recessive** | |
| --- | --- |
| **ADCK4** | AarF domain containing kinase 4 |
| **ARHGDIA** | Rho GDP dissociation inhibitor (GDI) alpha |
| **CD2AP** | CD2-associated protein |
| **CFH** | Complement factor H |
| **COQ2** | Coenzyme Q2 4-hydroxybenzoate, Polyprenyltransferase |
| **COQ6** | Coenzyme Q6 monooxygenase |
| **CRB2** | Crumbs homolog 2 |
| **CUBN** | Cubilin (intrinsic factor-cobalamin receptor) |
| **DGKE** | Diacylglycerol kinase, epsilon |
| **EMP2** | Epithelial membrane protein 2 |
| **FAT1** | FAT tumor suppressor homolog 1 |
| **ITGA3** | Integrin, alpha 3 (antigen CD49C, alpha 3 subunit of VLA-3 receptor) |
| **ITGB4** | Integrin, beta 4 |
| **KANK1** | KN motif and ankyrin repeat domain containing protein 1 |
| **KANK2** | KN motif and ankyrin repeat domain containing protein 2 |
| **KANK4** | KN motif and ankyrin repeat domain containing protein 4 |
| **LAMB2** | Laminin, β2 |
| **MTTL1** | Mitochondrially encoded tRNA leucine 1 |
| **MYO1E** | Homo sapiens myosin IE (MYO1E) |
| **NPHS1** | Nephrin |
| **NPHS2** | Podocin |
| **NUP93** | Nucleoporin 93 kDa |
| **NUP107** | Nucleoporin 107 kDa |
| **NUP205** | Nucleoporin 205 kDA |
| **PDSS2** | Prenyl (decaprenyl) diphosphate synthase, subunit 2 |
| **PLCE1** | Phospholipase C, epsilon 1 |
| **PTPRO** | Protein tyrosine phosphatase, receptor type, O |
| **SCARB2** | Scavenger receptor class B, member 2 |
| **SMARCAL1** | SWI/SNF related, matrix associated, actin dependent regulator of chromatin, subfamily a like 1 |
| **WDR73** | WD repeat domain 73 |
| **XPO5** | Exportin 5 |
| **Autosomal dominant** | |
| **ACTN4** | Actinin, alpha 4 |
| **ANLN** | Anillin, actin binding protein |
| **ARHGAP24** | Rho GTPase activating protein 24 |
| **INF2** | Inverted formin, FH2 and WH2 domain containing |
| **LMX1B** | LIM homeobox transcription factor 1, beta |
| **MYH9** | Myosin heavy chain 9 |
| **TRPC6** | Transient receptor potential cation channel, subfamily C, member 6 |
| **WT1** | Wilms tumor 1 |

**Table S4.** Gluten urine dipstick results performed during the camp (day 20).

|  | **Day 20** |
| --- | --- |
| Patient 01* | negative |
| Patient 02 | negative |
| Patient 03 | negative |
| Patient 04 | negative |
| Patient 06* | negative |
| Patient 07 | negative |
| Patient 08 | negative |
| Patient 09 | negative |
| Patient 10 | negative |
| Patient 11 | negative |
| Patient 12 | negative |
| Patient 14 | negative |
| Patient 15 | negative |
| Patient 16 | negative |
| Patient 17 | negative |
| Patient 18 | negative |

*Patients 1 and 6 were the responders

**Table S5.** Demographics and clinical features of patients with SRNS included in the study.

| **Patient no.** | **Age at Camp** | **Sex** | **Age at onset of the disease** | **Ethnicity** | **Histological diagnosis** | **Steroid treatment** | **Other IS treatment** | **History of allergy** | **Identified podocyte genetic mutation** | **Number of anti-HTN drugs** | **Previous remission** | **Clinical presentation of the disease** | | | **Complications** |
| --- | --- | --- | --- | --- | --- | --- | --- | --- | --- | --- | --- | --- | --- | --- | --- |
|  |  |  |  |  |  |  |  |  |  |  |  | HTN | He | NS |  |
| 01* | 2.2 | F | 1.3 | MR | MCD | Current | CsA | N | N | 1 | N | N | N | Y | Growth retardation  Frequent mild infections** |
| 02 | 6.5 | M | 4.3 | AA | MCD | Current | Tac, CsA, Ritux, | Y | N | 2 | PR | N | N | Y | Gain weight  Serious bacterial infection# |
| 03 | 6.0 | M | 2.7 | C | FSGS | Past | Tac, CsA, MPA, CYC | N | N | 3 | N | N | N | Y | Frequent mild infections, cataract,  hypothyroidism |
| 04 | 7.5 | M | 3.5 | C | FSGS | Past | Tac, CsA | Y | N | 1 | N | Y | Y | Y | Frequent mild infections |
| 06* | 9.8 | M | 9 | H | FSGS | Current | No | Y | N | 1 | CR | N | N | Y | Frequent mild infections |
| 07 | 9.5 | F | 7.7 | C | FSGS | Past | Tac, MPA | N | N | 2 | CR | N | Y | Y | Weight gain |
| 08 | 9.3 | F | 3.9 | MR | FSGS | Past | CsA, MPA, CYC, Ritux, IVIG | N | N | 1 | N | N | N | Y | Growth retardation,  Cataract  Frequent mild infections |
| 09 | 7.4 | F | 0.8 | C | MCD | Past | CsA | N | N | 1 | CR | Y | Y | Y | Frequent mild infections |
| 10 | 12.5 | F | 4.3 | C | MCD | Past | No | Y | N | 1 | N | N | N | Y | Frequent mild infections |
| 11 | 13.8 | F | 13.1 | MR | MCD | Past | Tac, MPA | Y | N | 1 | N | N | N | Y | Gain weight,  Serious bacterial infection |
| 12 | 14.4 | F | 13.2 | C | FSGS | Past | Tac, Ritux, IVIG | N | N | 1 | N | N | Y | Y | Serious bacterial infection |
| 14 | 15.3 | M | 12.5 | H | FSGS | Past | Tac, CsA, MPA, CYC | Y | N | 2 | N | Y | N | Y | Serious bacterial infection  Avascular hip necrosis  Weight gain, allergic drug reaction |
| 15 | 16.7 | M | 13.4 | H | FSGS | Past | CsA, MPA, CYC, Ritux | N | N | 2 | PR | N | N | Y | No |
| 16 | 18.9 | M | 7.3 | MR | MCD | Past | Tac, CsA, MPA, Ritux, IVIG | N | N | 1 | N | N | N | Y | Frequent mild infections, cataract, Ostopenia |
| 17 | 21.1 | F | 18 | AA | FSGS | Current | Tac | Y | N | 1 | PR | N | N | Y | Serious bacterial infection, weight gain |
| 18 | 12.2 | M | 11.2 | C | FSGS | Current | Tac, CsA, MPA, Ritux | N | N | 1 | PR | N | Y | Y | Cataract |

F, female; M, male; MR, multi-racial; AA, African-American, C, Caucasian; H, Hispanic; MCD, minimal change disease; FSGS, focal and segmental glomerulosclerosis; CsA, cyclosporine; Tac, tacrolimus, Ritux, rituximab, MPA, mycophenolic acid; CYC, cyclophosphamide; IVIG, intravenous immunoglobulin; N, no; Y, yes, HTN, hypertension, PR, partial remission; CR, complete remission; He, hematuria; NS nephrotic syndrome

* Patients 1 and 6 were the responders

**Frequent mild infections include gastroenteritis, urinary tract infection, skin infection, flu, rhinitis

#Serious bacterial infections include sepsis, pneumonia, endocarditis

## Supplementary Figures


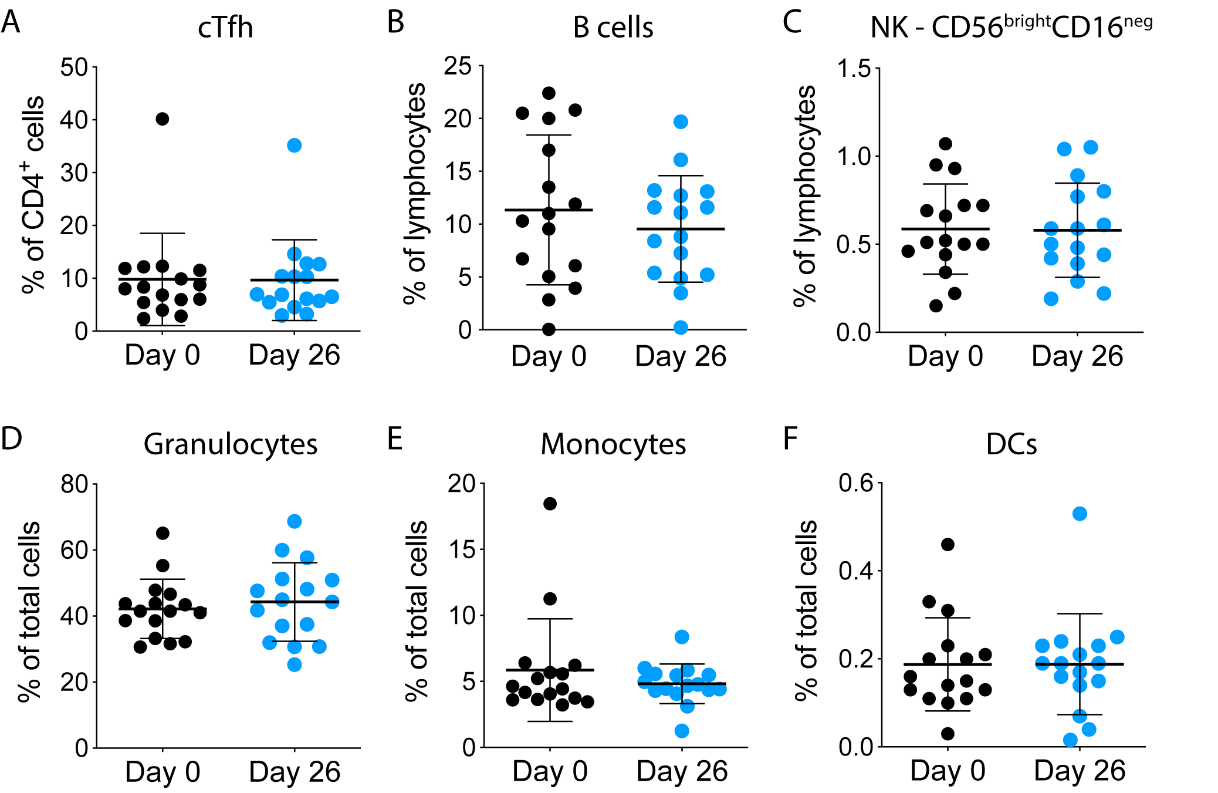


**Supplementary Figure 1.** **Changes in immune cells populations by flow cytometry after GF/DF dietary intervention.** Fresh blood samples obtained from children at day 0 and day 26 during the camp were processed by flow cytometry. Wilcoxon matched pair test was performed in order to assess differences in the immune cell subpopulations before and after dietary intervention.

cTFh, circulating T follicular helper; NK, natural killer; DCs, dendritic cells

**
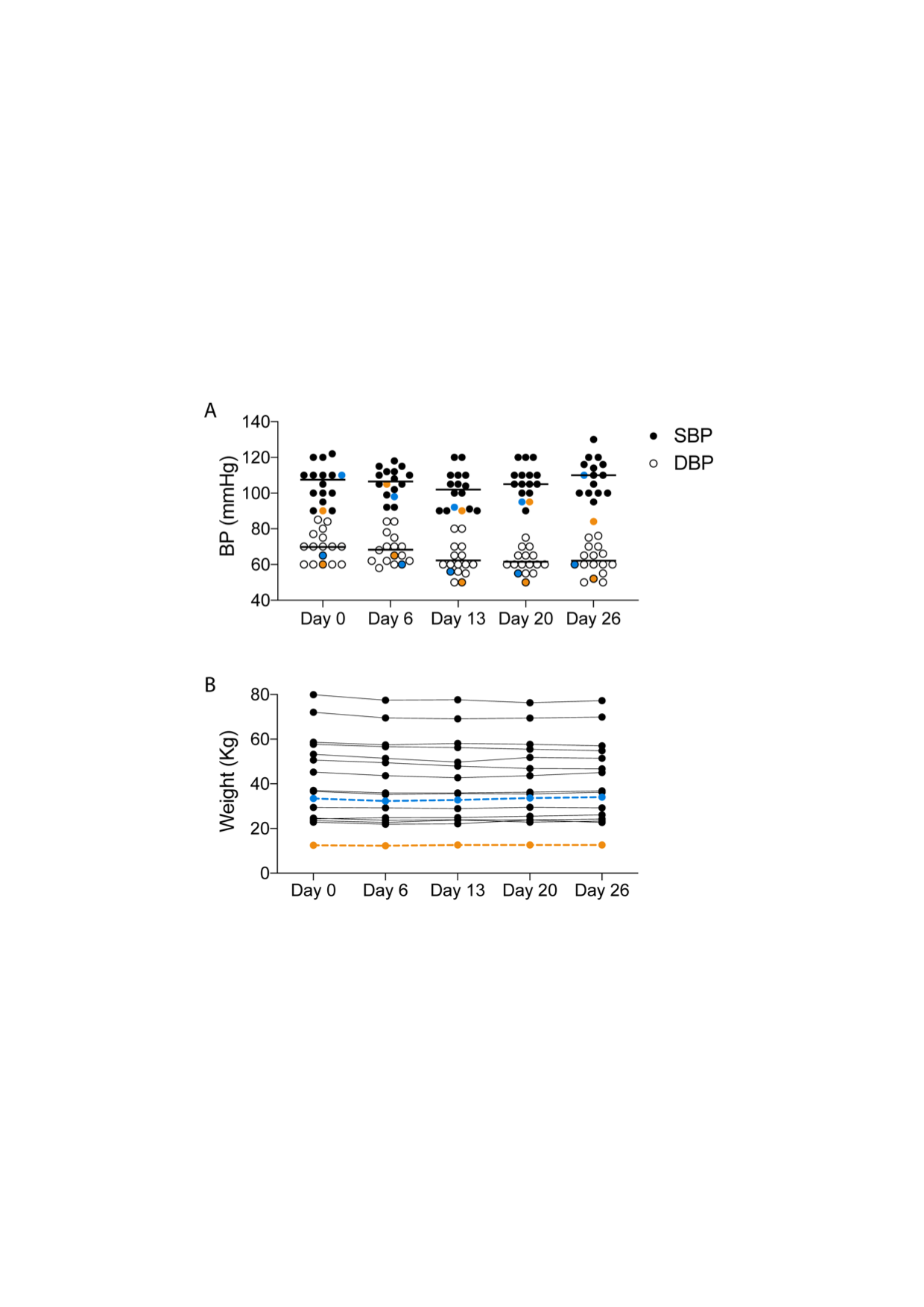
**

**Supplementary Figure 2. Blood pressure and weight evolution in all participants during the study period.** Responders are highlighted in colors (blue and orange).

BP, blood pressure; SBP, systolic blood pressure; DBP, diastolic blood pressure

**
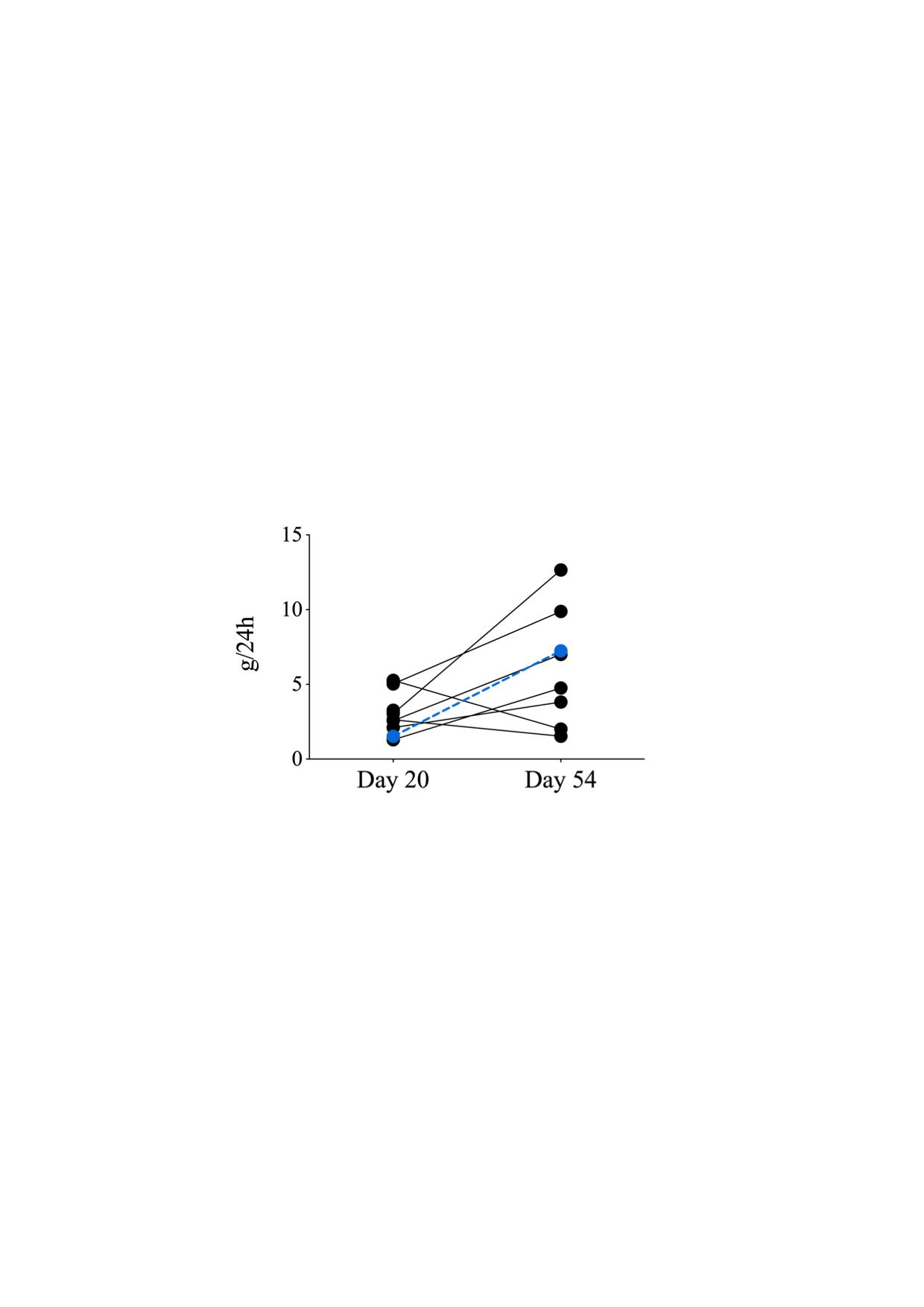
**

**Supplementary Figure 3. Mean of salt intake during camp and after that at home.** Salt intake was calculated by measurement of 24h sodium urine excretion. One of the responders (patient 01, orange) was unable to collect 24-h urine due to her age.
